# Epizootic shell disease induces systemic transcriptomic shifts in *Homarus americanus*, characterized by increased shell degradation and impaired energy metabolism across tissues

**DOI:** 10.3389/fphys.2025.1642696

**Published:** 2025-08-01

**Authors:** Minseo Kim, Dianjun Cao, Vincent Cavaleri, Kyudong Han, Seyoung Mun, Soo Jin Jeon

**Affiliations:** ^1^Department of Microbiology, College of Bio-convergence, Dankook University, Cheonan, Republic of Korea; ^2^Lewyt College of Veterinary Medicine, Long Island University, Brookville, NY, United States; ^3^Division of Marine Resources, New York State Department of Environmental Conservation, East Setauket, NY, United States; ^4^Smart Animal Bio Institute, Dankook University, Cheonan, Republic of Korea; ^5^Center for Bio Medical Engineering Core Facility, Dankook University, Cheonan, Republic of Korea; ^6^Department of Cosmedical and Materials, Dankook University, Cheonan, Republic of Korea

**Keywords:** American lobster, epizootic shell disease, transcriptome analysis, antilipopolysaccharide factor, chitinase, acetyl-coenzyme A transporter, heat shock protein, polymicrobial infection

## Abstract

Epizootic shell disease (ESD) is characterized by shell erosion, pitting, and melanization in the American lobster (*Homarus americanus*) and is associated with a polymicrobial infection. The disease is multifactorial, with several contributing factors such as rising water temperatures and environmental pollution, which may facilitate bacterial invasion and increase host susceptibility. In a previous study, we found that the microbiome composition of the carapace in lobsters with ESD differed from that of healthy individuals, with ESD-associated bacteria enriched in the green gland and testis. However, the effects of bacterial infection on internal organs have not been clearly identified. In this study, we investigated the effects of ESD on four major tissues of the lobster (testis, intestine, hepatopancreas, and green gland) using transcriptomic analysis. A total of 564 genes were differentially expressed in the testis, 105 in the intestine, 333 in the hepatopancreas, and 112 in the green gland. The expression of the *anti-lipopolysaccharide factor* gene was increased in all tissues, indicating a systemic immune response to bacterial infection. Notably, *chitinase* genes involved in chitin degradation were upregulated, while the *acetyl-coenzyme A transporter 1-like* gene related to energy metabolism was significantly downregulated in the testis. In the intestine, expression of *phosphoenolpyruvate carboxykinase cytosolic [GTP]* and *cytochrome P450* genes, which are involved in gluconeogenesis and xenobiotic metabolism, respectively, was reduced. The hepatopancreas showed decreased expression of *hemocyanin* genes, which play key roles in oxygen transport and immune defense in crustaceans. The green gland exhibited reduced expression of *heat shock proteins* involved in the cellular stress response, *organic cation transporter proteins* that mediate the excretion of organic cations, and *UDP-xylose and UDP-N-acetylglucosamine transporters* required for glycosylation and chitin biosynthesis. Together, these transcriptional changes suggest that ESD may compromise physiological functions such as immune defense, energy metabolism, and stress response, while promoting chitin degradation and cuticle remodeling in response to shell infection. This study revealed tissue-specific transcriptomic responses to ESD in the American lobster, providing a foundation for elucidating the molecular mechanisms underlying disease progression.

## 1 Introduction

The American lobster (*Homarus americanus*) is distributed throughout the coastal waters of the western North Atlantic. Long Island Sound, a large estuary between Connecticut and Long Island, was one of the largest lobster habitats in the United States until the late 1990s. Over the past 2 decades, however, the region has experienced a substantial decline in lobster populations (Long Island Sound Study, 2024). This decline has been associated with the increasing prevalence of epizootic shell disease (Castro et al., 2012). First reported in 1996, disease prevalence remains moderate to high, particularly in the eastern Long Island Sound (Castro and Somers, 2012; Tanaka et al., 2017).

Epizootic shell disease (ESD) is characterized by erosion, pitting, and melanization of the shell, resulting from bacterial infection (Smolowitz et al., 2005). Elevated water temperature is a key contributing factor, as high temperatures and other stressors can disrupt lobsters’ homeostasis, molting, and resistance to infection, increasing their susceptibility to ESD (Dove et al., 2005; Ishaq et al., 2022; Lorenzon et al., 2007). The genus *Aquimarina* has been consistently identified as the primary causative pathogen (Ooi et al., 2020; Quinn et al., 2017; Quinn et al., 2012), although various rod-shaped bacteria colonize shell lesions (Davies et al., 2014). In addition to shell infection, carapace bacteria have been detected in internal organs (Schaubeck et al., 2023), indicating the possibility of systemic infection. In lobsters with ESD, the expression of the ecdysteroid receptor and cytochrome P450 is increased in the hepatopancreas, while the expression of arginine kinase is downregulated in the muscle (Tarrant et al., 2010; Tarrant et al., 2012). Additionally, female lobsters with ESD show elevated levels of macroglobulin, a protease inhibitor related to the innate immune system, in the ovary (Tarrant et al., 2010). Male lobsters with ESD exhibit testicular abnormalities, resulting in over 50% of spermatozoa being nonviable (Taylor et al., 2021). These findings suggest that ESD is associated with systemic infection, which may impair immunity, metabolism, and reproductive function.

Transcriptome analysis provides a powerful approach for unraveling the complex biological alterations and pathogenic mechanisms underlying disease, enabling the identification of key molecular pathways. Under simulating ocean warming conditions, lobsters exposed to elevated temperatures after the larval stage showed up to a 7.1-fold increase in the expression of metabolism-related transcripts, suggesting that failure to meet rising energy demands could lead to increased mortality from disease and starvation (Harrington et al., 2020). In addition, reduced expression of chitin-related and pseudohemocyanin genes in the hepatopancreas has been linked to the progression of impoundment shell disease (Argenta et al., 2024). However, research on transcriptomic changes across internal tissues during ESD remains limited.

We have previously revealed that the carapace microbiota of lobsters with ESD harbors a high abundance of *Aquimarina, Halocynthiibacter*, and *Tenacibaculum* (Schaubeck et al., 2023). These carapace bacteria have also been detected in internal organs, including the green gland, hepatopancreas, intestine, and testis, and appear to be more common in lobsters with ESD compared to healthy lobsters. Therefore, it is hypothesized that ESD-associated bacteria translocate from the carapace to internal organs, where they disrupt local tissue homeostasis, leading to systemic dysfunction and disease progression. To test this hypothesis, this study aimed to identify tissue-specific molecular responses in lobsters affected by ESD.

## 2 Materials and methods

### 2.1 Lobster sampling

American lobsters were collected in August 2020 from eastern Long Island Sound and approximately 50 miles south of Montauk, in collaboration with local lobstermen and the New York State Department of Environmental Conservation. Carapace lengths ranged from 24.0 to 28.5 cm, and body weights ranged from 540.5 to 874.6 g (Supplementary Table S1). To prevent cross-contamination, lobsters were placed in a cooler on ice and sorted into two groups based on the presence (ESD, n = 37) or absence (HTH, n = 33) of shell lesions, then immediately transported to the laboratory. Following ice chilling for 30 min to minimize mobility and stress, the lobsters were dissected to collect various tissues, including the testis, intestine, hepatopancreas (HP), and green gland (GG). The collected tissues were gently washed with phosphate-buffered saline (PBS, pH 7.4) and stored in RNAlater stabilization solution (Qiagen) at −80°C until RNA extraction.

### 2.2 RNA sequencing

Total RNA was extracted from 10 mg of homogenized tissues using the RNeasy® Plus Mini Kit (Qiagen) following the manufacturer’s instructions. RNA quantity and purity were evaluated with a NanoDrop OneC microvolume UV-Vis spectrophotometer (Thermo Scientific). Purity was assessed based on the A260/A280 ratio, with values between 2.0 and 2.2, indicating acceptable RNA quality. The RNA samples were stored at −80°C until use in RNA sequencing (RNA-seq). The frozen RNA samples were shipped on dry ice to Genewiz (South Plainfield, NJ) for RNA-seq. The RNA-seq workflow comprised Poly A selection-based mRNA enrichment, mRNA fragmentation, and ramdom priming with subsequent first- and second-strand complementary DNA (cDNA) synthesis. End-repair 5′phosphorylation and adenine nucleotide (dA)-tailing were then performed. Finally, adaptor ligation, polymerase chain reaction (PCR) enrichment, and sequencing using the Illumina HiSeq 2,500 platform with 2 × 150 bp paired-end reads were performed. Quality controls and basic statistical methods were performed using FastQC (https://www.bioinformatics.babraham.ac.uk) to remove low quality reads as follows: Reads containing more than 10% skipped bases (labeled as ‘N'), sequencing reads containing more than 40% of bases with a quality score of less than 27, and average quality score (<27). Quality distributions of nucleotide, GC content, PCR duplicate characteristics, and k-mer sequencing data frequency were calculated.

### 2.3 Transcript read alignment

Sequencing reads were aligned to the GMGI_Hamer_2.0 *H. americanus* genome using STAR software (v.2.7.10a) with default parameters (accession: GCA_018991925.1, https://www.ncbi.nlm.nih.gov/data-hub/genome/GCF_018991925.1/). Then, gene expression was quantified using RSEM (v.1.3.3) software with default parameters and annotation from *H. americanus* Ensembl genes.

### 2.4 Analysis process for differentially expressed genes

Uniquely mapped read pairs were used for differentially expressed genes (DEGs) analysis. Gene expression counts were generated using RSEM (v.1.3.3) and normalized to transcripts per million (TPM). A total of 70 samples were initially used for RNA-seq. To ensure consistency, samples with a mapping rate below 65% and those identified as outliers were excluded. As a result, 53 samples were retained for downstream analysis. The final dataset consisted of the following tissues: testis (HTH = 6; ESD = 6), intestine (HTH = 6; ESD = 8), HP (HTH = 5; ESD = 5), and GG (HTH = 9; ESD = 8). All four tissue types used for RNA-seq were collected from the same individual lobsters (Supplementary Table S2). Gene expression differences between the two groups were considered statistically significant if |Log_2_ (fold change)| ≥ 1 and *p*-value <0.05 using the DESeq2 (v.1.34.0) in R package (v.4.4.1) (Love et al., 2014). A volcano plot was generated using the EnhancedVolcano package (Blighe et al., 2021), and a heatmap was generated using Pheatmap (v.1.0.12).

### 2.5 Gene Ontology function enrichment analysis

Gene Ontology (GO) term enrichment analysis was conducted using g:profiler (Raudvere et al., 2019). All genes with known statistical domain coverage were included, and multiple testing correction was applied using the Benjamini–Hochberg method with a significance threshold of 0.05. Gene annotations were obtained from Ensembl metazoan, which provides genomic data for non-vertebrate metazoan species including crustaceans (Howe et al., 2020). Pathway visualization was carried out using ggplot2 (v.3.5.2) in R.

### 2.6 Kyoto Encyclopedia of Genes and Genomes analysis

Kyoto Encyclopedia of Genes and Genomes (KEGG) pathway analysis was performed to understand various biological functions and pathways in disease states. KEGG enrichment analysis of DEGs by tissue was performed using the clusterProfiler (v.4.12.6) in R (Kanehisa and Goto, 2000).

### 2.7 Selection of representative genes for expression analysis

To explore gene expression patterns, we visualized 16 DEGs (four from each organ) related to gluconeogenesis, innate immune system, and molting based on GO and KEGG pathway analysis. Gene expression values for each sample were converted to log_2_ (TPM +0.1) and visualized as violin plots using GraphPad Prism 10.

### 2.8 Validation of quantitative real-time polymerase chain reaction (qRT-PCR)

First-strand cDNA was synthesized from RNA samples using the Verso cDNA Synthesis Kit (Thermo Scientific) with a 3:1 blend of random hexamers and anchored oligo-dT. The synthesis conditions were as follows: 42°C for 30 min, 50°C for 30 min, 95°C for 2 min, followed by holding at 4°C. The synthesized cDNAs were stored at −80°C until further use. cDNA amplification was performed using gene-specific primers targeting *acidic mammalian chitinase-like* (LOC121854211), *probable chitinase 2* (LOC121878393), *chitinase-3-like protein 1* (LOC121860919), and *acetyl-coenzyme A transporter 1-like* (LOC121868265). To verify the functionality of the primers used in qRT-PCR, preliminary amplification was carried out using the T100 thermal cycler (Bio-Rad) at the Bio-medical engineering Core Facility of Dankook University. Following validation, qRT-PCR was performed using the CFX96 Duet Real-Time PCR System (Bio-Rad). All reactions were performed using 1 µL of cDNA, 10 pmol of primers and 2X Taq Pro Universal SYBR qRT-PCR Master Mix in a final volume of 20 µL. The PCR reaction started with a heating step at 95°C for 3 min, followed by 40 cycles of denaturation at 95°C for 10 s, annealing at the optimum temperature for 10 s, and extension at 72°C for 15 s. A melt curve analysis was performed from 62°C to 95°C, with a ramp rate of 0.5°C per minute. Gene expression was normalized to the expression of *glycerol aldehyde-3-phosphate dehydrogenase* (GAPDH), a housekeeping gene. A positive qRT-PCR reaction was detected based on the accumulation of fluorescent signals, and the cycle threshold (CT; the number of cycles required for the fluorescent signal to cross a fixed threshold) is inversely proportional to the amount of target nucleic acid in the sample. Relative gene expression was calculated using the 2^−ΔΔCt^ value of each sample, and qRT-PCR was performed using five technical replicates per group for each cDNA sample. The primer sequences used are listed in Supplementary Table S1.

### 2.9 Statistical analysis

The pairwise Pearson correlation coefficient was computed using log-transformed TPM values for all expressed genes. The correlation coefficients were calculated using the base R function and visualized as a correlation matrix using the ggcorrplot (v.0.1.4.1) in R. Principal component analysis (PCA) was performed to assess sample distribution patterns using the built-in prcomp () function in R, and the results were visualized with ggplot2. Permutational multivariate analysis of variance (PERMANOVA) was conducted to statistically evaluate group differences by tissue type and disease status. TPM values were log_2_-transformed, followed by a Hellinger transformation, and then used to calculate a Euclidean distance matrix. PERMANOVA was performed with 10,000 permutations using the adonis function from the vegan (v.2.6–10) in R. TPM distributions across all samples were visualized using log_10_-transformed values, and density plots were generated with ggplot2 in R. The statistical significance of KEGG pathway enrichment was determined based on the Benjamini–Hochberg adjusted *p*-value (FDR <0.05). To validate RNA-seq results, qRT-PCR was performed, and relative gene expression levels (fold changes) were calculated using the 2^−ΔΔCt^ method. Statistical comparisons between the ESD and HTH groups were conducted on ΔCt values using a Student’s t-test. Significance levels were set at p* <0.05 and p** <0.01.

## 3 Results

### 3.1 Clinical symptom and visual characteristics of epizootic shell disease

**FIGURE 1:**
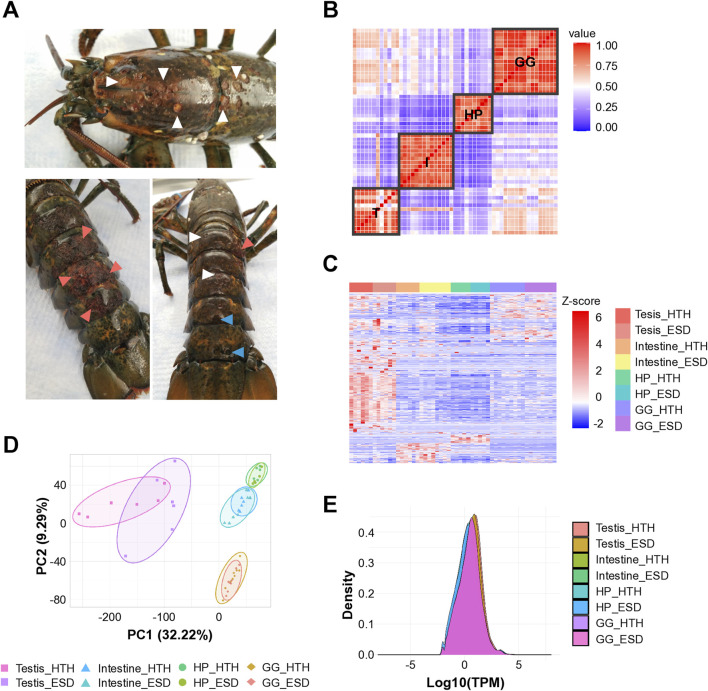
Sample collection and RNA sequencing quality assessment. **(A)** American lobsters with epizootic shell disease (ESD). White triangles indicate pits; pink triangles indicate erosion; and blue triangles denote both pits and melanin deposition. **(B)** Pairwise correlation analysis showing tissue-specificity. **(C)** Heatmap of differentially expressed genes across tissues. Each column represents a gene, and each row indicates a sample. T: Testis; I: Intestine; HP: Hepatopancreas; GG: Green gland. **(D)** Principal component analysis showing tissue-specific transcriptional profiles (PERMANOVA, *P* < 0.0001). **(E)** Density plot showing consistent expression distributions across tissue samples. HTH: Healthy lobsters; ESD: Lobsters with ESD.

American lobsters were collected from eastern Long Island Sound in August 2020. Lobsters with ESD exhibited carapace lesions characterized by pitting, erosion, and melanization (Figure 1A). White triangles indicate pits, pink triangles indicate erosion of the carapace, and blue triangles denote both pits and melanin deposition.

### 3.2 Transcriptome sequencing analysis

The Illumina HiSeq 2,500 instrument was used, and the read length was 150 bp. The number of uniquely mapped reads averaged 18,007,700 out of 24,950,208 total reads, with a mapping rate of 72.25% against the *H*. *americanus* reference genome (Supplementary Table S2). We performed pairwise correlation analysis based on the total gene expression values across samples to determine the reproducibility of technical replicates and differences in gene expression (Figure 1B). Consequently, robust correlations emerged across multiple tissues, suggesting tissue-dependent responses, with the testis exhibiting pronounced variability among lobsters. We visualized the DEGs from all organs using a heatmap and found that the gene expression differed by tissues and groups (Figure 1C). Notably, the testis showed clusters of genes that were strongly upregulated (red) or downregulated (blue) compared to other tissues, highlighting significant transcriptional changes compared to other tissues. PCA was performed to measure the distance between samples and confirmed significant separation by tissue (Figure 1D; PERMANOVA, p < 0.0001). Density plots showed overlapping curves across samples, indicating that the RNA-seq data were consistent in quality, reproducible across replicates, and comparable across tissue types (Figure 1E).

### 3.3 Differentially expressed genes analysis

**FIGURE 2:**
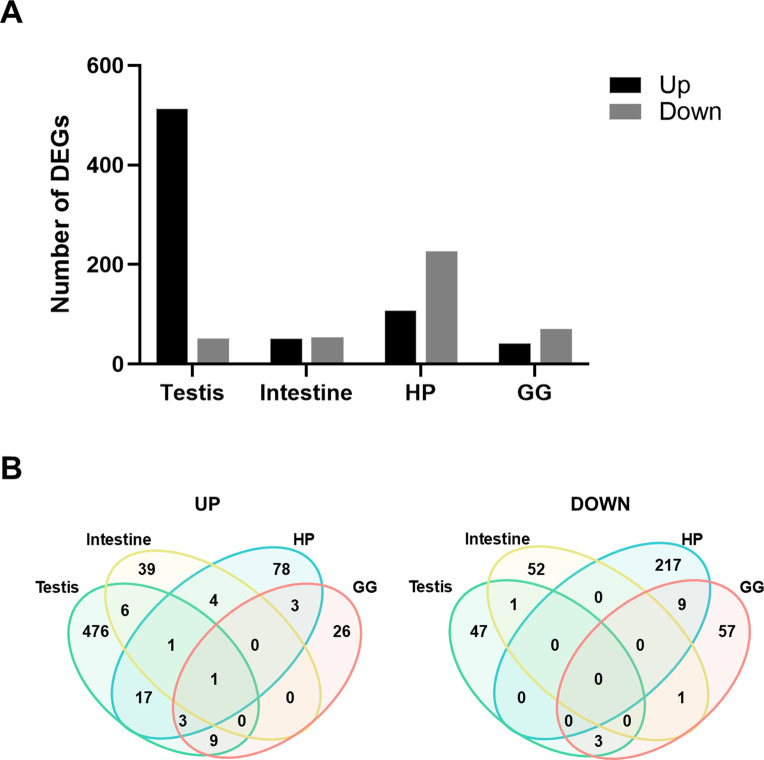
Differentially expressed genes (DEGs) across tissues in lobsters with epizootic shell disease (ESD). **(A)** Number of DEGs identified in each tissue. HP: Hepatopancreas; GG: Green gland. **(B)** Venn diagrams showing the number of shared upregulated and downregulated DEGs among tissues. The *anti-lipopolysaccharide factor* gene was consistently upregulated across all tissues in lobsters with ESD.

We analyzed gene expression across tissues using DESeq2 to identify DEGs in the ESD group relative to the HTH group. In the testis, 513 genes were upregulated and 51 were downregulated in ESD compared to HTH. In the intestine, 51 genes were upregulated and 54 were downregulated. The HP exhibited 107 upregulated and 226 downregulated genes, while the GG showed 42 upregulated and 70 downregulated genes (Figure 2A). Among these DEGs, the *anti-lipopolysaccharide factor* gene (LOC121856986) was consistently upregulated across all tissues examined (Figure 2B). The testis, HP, and GG shared elevated expression of the *soma ferritin-like* (LOC121859498) and two *anti-lipopolysaccharide factor-like* genes (LOC121860186 and LOC121860188). Additionally, the testis, intestine, and HP shared increased expression of the *lipase three* gene (LOC121863816). The HP and GG demonstrated a consistent decrease in the expression of nine genes, including *heat shock 70 kDa protein* (LOC121855754, LOC121855757, and LOC121870242), *la-related protein 6* (LOC121859532), *cytochrome P450 2L1-like* (LOC121876874), *D-β hydroxybutyrate dehydrogenase* (LOC121866996 and LOC121878121), *estradiol 17-β-dehydrogenase 2* (LOC121878174), and an uncharacterized gene (LOC121880200).

### 3.4 Gene Ontology of differentially expressed genes

**FIGURE 3:**
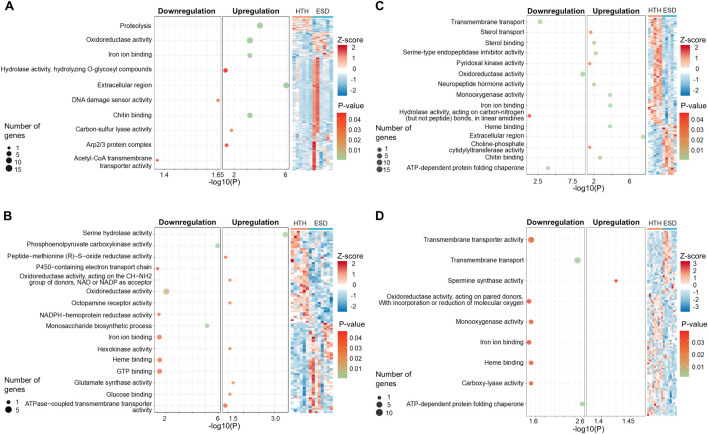
Gene ontology (GO) enrichment analysis of differentially expressed genes (DEGs) in lobsters with epizootic shell disease (ESD) compared to healthy individuals. **(A)** Testis, **(B)** Intestine, **(C)** Hepatopancreas, **(D)** Green gland. Each dot represents a significantly enriched GO term. Dot size indicates the number of associated DEGs, and color represents statistical significance (adjusted *p*-value). HTH: Healthy lobsters; ESD: Lobsters with ESD. The heatmap illustrates the expression patterns of DEGs. Expression values were normalized using Z-score. Red and blue indicate upregulation and downregulation, respectively.

All DEGs identified in the ESD group compared to the HTH group were analyzed for functional enrichment based on the GO terms. In the testis, the heatmap revealed distinct clustering of samples by health status (HTH vs ESD), although some variability among individuals was observed (Figure 3A). Upregulated pathways were associated with the GO terms chitin binding (GO:0008061), hydrolase activity, hydrolyzing O-glycosyl compounds (GO:0004553), proteolysis (GO:0006508), and oxidoreductase activity (GO:0016491). The chitin binding pathway was enriched with 13 DEGs, and six genes were predicted to encode chitinase (Supplementary Table S3). In contrast, the pathways for acetyl-CoA transmembrane transporter activity (GO:0008521) and DNA damage sensor activity (GO:0140612) were downregulated. In the intestine (Figure 3B), the most enriched upregulated pathways included serine hydrolase activity (GO:0017171) and ATPase-coupled transmembrane transporter activity (GO:0042626), while the most significantly downregulated pathways were phosphoenolpyruvate carboxykinase activity (GO:0004611) and monosaccharide biosynthetic process (GO:0046364). Additionally, genes involved in metabolism and redox balance were downregulated (Supplementary Table S4). In the HP (Figure 3C), the upregulated pathways included monooxygenase activity (GO:0004497), heme binding (GO:0020037), and chitin binding (GO:0008061), while the downregulated pathways were oxidoreductase activity (GO:0016491) and transmembrane transport (GO:0055085). Notably, ten downregulated genes associated with oxidoreductase activity (GO:0016491) were identified as hemocyanin A and B. The pathway related to ATP-dependent protein folding chaperone primarily contained six genes belonging to the heat shock protein 70 kDa (Supplementary Table S5). In the GG (Figure 3D), the spermine synthase activity (GO:0016768) pathway was upregulated, while transmembrane transport (GO:0055085) and ATP-dependent protein folding chaperone (GO:0140662) pathways were downregulated. Consistent with the pattern observed in the HP, heat shock protein showed decreased expression (Supplementary Table S6).

### 3.5 Kyoto Encyclopedia of Genes and Genomes (KEGG) pathway analysis

**FIGURE 4:**
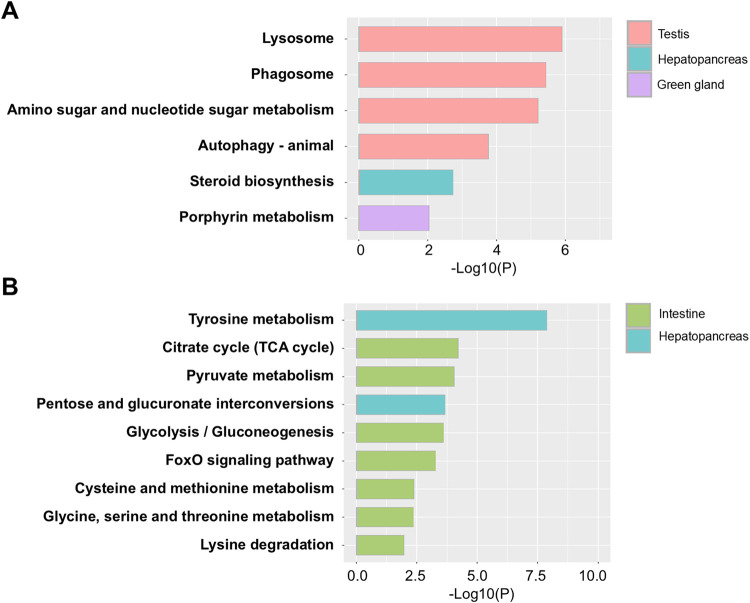
Kyoto Encyclopedia of Genes and Genomes (KEGG) pathway analysis of differentially expressed genes (DEGs) in lobsters with ESD. **(A)** Enriched KEGG pathways associated with upregulated genes. **(B)** Enriched KEGG pathways associated with downregulated genes. The y-axis represents pathway names, and the x-axis indicates statistical significance (*p*-values).

KEGG pathway analysis was performed to identify metabolic alterations associated with ESD across lobster tissues. In the testis, four KEGG pathways were significantly enriched in the ESD group. The most significantly increased pathways (Figure 4A) included: (1) lysosome (hame04142), (2) phagosome (hame04145), (3) amino sugar and nucleotide sugar metabolism (hame00520), and (4) autophagy - animal (hame04140). These enrichments may indicate activation of cellular degradation pathways and immune defense, along with increased metabolic activity to meet elevated energy demands. In the HP, the steroid biosynthesis pathway (hame00100) was significantly increased, which may reflect increased synthesis of molting- and reproduction-related hormones. In the GG, the porphyrin metabolism pathway (hame00860) was significantly upregulated, suggesting a potential increase in demand for iron metabolism and detoxification in response to bacterial infection. In contrast, downregulated pathways were primarily observed in the HP and intestine (Figure 4B). In the HP, tyrosine metabolism (hame00350) and pentose and glucuronate interconversions (hame00040) were significantly decreased, suggesting dysregulation of amino acid metabolism and detoxification pathways. In the intestine, downregulated genes were more prominent, with seven KEGG pathways enriched: (1) citrate cycle (TCA cycle) (hame00020), (2) pyruvate metabolism (hame00620), (3) glycolysis/gluconeogenesis (hame00010), (4) FoxO signaling pathway (hame04068), (5) cysteine and methionine metabolism (hame00270), (6) glycine, serine and threonine metabolism (hame00260), and (7) lysine degradation (hame00310). These findings suggest that ESD may be associated with metabolic suppression in the intestine, potentially leading to reduced energy production and impaired tissue function. Detailed results of the KEGG enrichment analysis are provided in Supplementary Table S7.

### 3.6 Expression analysis of selected target genes

**FIGURE 5:**
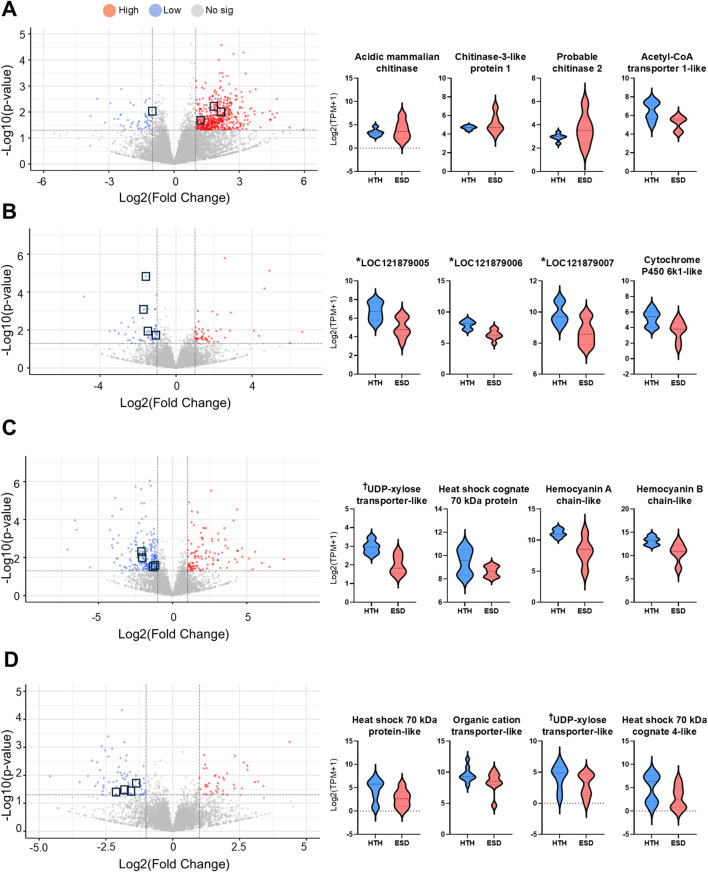
Distribution of differentially expressed genes (DEGs) and expression of key genes across tissues. **(A)** Testis, **(B)** Intestine, **(C)** Hepatopancreas, and **(D)** Green gland. Volcano plots display DEGs, with the y-axis representing -log_10_ (*p*-value) and the x-axis showing log_2_ (fold change). Upregulated genes are shown in red, downregulated genes in blue, and non-significant genes in gray. Violin plots illustrate the expression levels of selected key genes. The y-axis indicates log_2_ (TPM +1), and the x-axis indicates sample groups. The horizontal line within each violin plot represents the median expression level. ^*^LOC121879005, ^*^LOC121879006, and ^*^LOC121879007: annotated as phosphoenolpyruvate carboxykinase, cytosolic [GTP]-like enzymes involved in gluconeogenesis. ^†^UDP-xylose transporter-like: annotated as UDP-xylose and UDP-N-acetylglucosamine transporter involved in glycosylation pathways.

We selected 16 genes involved in glucose biosynthesis (energy metabolism), immune function, and the chitin synthesis pathway to further examine differential expression between the ESD and HTH groups. In the testis of the ESD group, three chitinase-related genes—*acidic mammalian chitinase-like* (LOC121854211), *chitinase-3-like protein 1* (LOC121860919), and *probable chitinase 2* (LOC121878393) — were significantly increased, while *acetyl-CoA transporter-1* (LOC121868265) was decreased (Figure 5A). In the intestine, all three genes from the *phosphoenolpyruvate carboxykinase cytosolic [GTP]* (LOC121879005, LOC121879006, and LOC121879007), as well as *cytochrome P450 6k1-like* (LOC121865918), were decreased in the ESD group (Figure 5B). In the HP, *UDP-xylose and UDP-N-acetylglucosamine transporter-like* (LOC121853811), *heat shock cognate 70 kDa protein* (LOC121862092), *hemocyanin A chain-like* (LOC 121877422), and *hemocyanin B chain-like* (LOC121863701) were decreased in the ESD group (Figure 5C). Similarly, in the GG, several genes showed decreased expression, including *heat shock protein 70 kDa protein-like* (LOC121855757), *heat shock 70 kDa protein cognate 4-like* (LOC121870242), *UDP-xylose and UDP-N-acetylglucosamine transporter-like* (LOC121869218), and *organic cation transporter protein-like* (LOC121863009) (Figure 5D). These results demonstrate tissue-specific molecular responses to ESD, characterized by enhanced chitinase activity in the testis and suppressed metabolic and stress-related pathways in the intestine, HP, and GG.

### 3.7 Validation of quantitative reverse transcription polymerase chain reaction (qRT-PCR)

**FIGURE 6:**
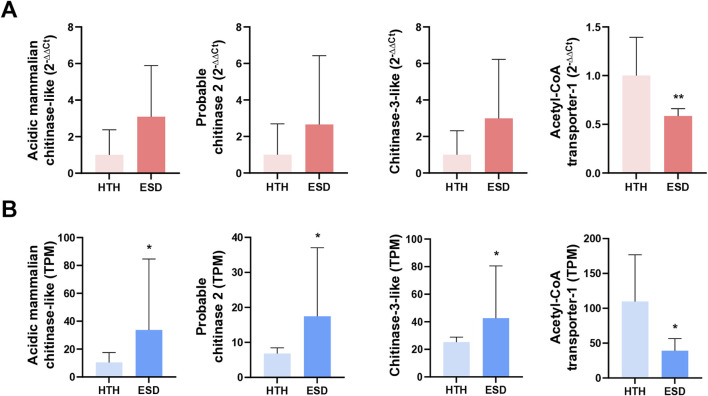
Validation of expression patterns for selected differentially expressed genes (DEGs) in the testis. **(A)** Relative expression levels measured by qRT-PCR, presented as fold change using the 2^−ΔΔCt^ method. Statistical analyses were performed on ΔCt values using an unpaired Student’s t-test. **(B)** Transcript abundance from RNA-seq, expressed as transcripts per million (TPM). Statistical significance was assessed using the Wald test. The genes analyzed include *acidic mammalian chitinase-like* (LOC121854211), *probable chitinase 2* (LOC121878393), *chitinase-3-like* (LOC121860919), and *acetyl-CoA transporter-1* (LOC121868265). Data represent mean values, and error bars indicate standard deviation (SD) based on biological replicates (n = 5). *p < 0.05; **p < 0.01.

We performed qRT-PCR to confirm the expression levels of three chitinase genes and the *acetyl-coenzyme A transporter 1-like* gene in the testis of the ESD and HTH groups (Figure 6A). These genes were selected based on enrichment on gene expression and functional relevance to chitin metabolism and immune response pathways. qPCR validation was performed using the same testis samples as those used for RNA-seq. Expression of the chitinase genes, including *acidic mammalian chitinase*, *chitinase-2*, and *chitinase-3,* increased by 3.09-fold (*p* = 0.09), 2.66-fold (*p* = 0.28), and 2.99-fold (*p* = 0.11), respectively, in the ESD group compared to the HTH group. Although these changes were not statistically significant, the qRT-PCR results confirmed the gene expression patterns observed in the RNA-seq data (Figure 6B). In contrast, expression of the *acetyl-coenzyme A transporter 1-like* gene was significantly decreased by 0.59-fold (*p* < 0.01) in the ESD group relative to the HTH group. These findings suggest that ESD may involve dysregulation of genes related to chitin metabolism and energy transport.

## 4 Discussion

Our study is the first to characterize gene expression changes in the internal organs of the American lobster with ESD using high-throughput sequencing technology. Among 1,114 DEGs, the *anti-lipopolysaccharide factor-like* gene was upregulated in all tested organs of lobsters with ESD. Anti-lipopolysaccharide factors (ALF) such as *ALFHa-1* and *ALFHa-2* have been identified in *H. americanus*, and notably, the expression of the *ALFHa-1* gene has been shown to increase in the gills following exposure to *Vibrio fluvialis* (Beale et al., 2008). ALF function as key components of the innate immune system by binding to lipopolysaccharides (LPS) in the outer membrane of Gram-negative bacteria, thereby neutralizing endotoxins and increasing permeability of bacterial membrane (Chaby, 2004). Therefore, the upregulation of ALF-like gene expression in the testis, intestine, HP, and GG indicates an innate immune response to Gram-negative bacterial invasion and supports the presence of systemic infection associated with ESD (Schaubeck et al., 2023).

We identified the chitin binding pathway as the most enriched differentially expressed gene pathway in the testis, with chitinase genes significantly upregulated within this pathway. Chitin is a homopolymeric carbohydrate composed of repeating N-acetyl-β-D-glucosamine (GlcNAc) units (Rowley and Coates, 2023). This compound is primarily found in the shells of crustaceans and the cell walls of fungi, providing the rigidity and mechanical strength needed to support the structure and shape of the organism (Zhang et al., 2021). Crustaceans such as shrimp and crabs contain the highest levels of chitin, which is highly associated with their growth and development (Zhang et al., 2021). Chitinase hydrolyzes chitin at intermediate regions to produce GlcNAc oligomers and is known to be induced in shrimp shells before molting (Rocha et al., 2012). Therefore, the observed upregulation of chitinase in lobsters with ESD may represent a molting-like response induced by shell infection. Although the upregulation of chitinase expression in the testis has not been widely reported, it is plausible that systemic immune and stress responses induced by ESD may also affect reproductive tissues. Chitinases are known to be involved not only in chitin degradation but also in immune defense and tissue remodeling across various tissues (Niu et al., 2018; Liu et al., 2019; Muthukrishnan et al., 2019; Lee et al., 2011). Therefore, the increased expression of chitinase observed in the testis may reflect a localized immune activation or structural remodeling of damaged tissue. Further investigation is required to clarify the functional role of chitinase in testicular physiology, particularly under disease conditions. In contrast, we observed significant downregulation of the acetyl-CoA transmembrane transport pathway in the testis. Acetyl-CoA is directly involved in major cellular processes, including energy metabolism, mitosis, and autophagy (Pietrocola et al., 2015), and also serves as a key substrate for chitin biosynthesis (Abo Elsoud and Kady, 2019). Additionally, we identified downregulation of genes encoding the transport protein responsible for the translocation of UDP-N-acetylglucosamine, a key precursor in the chitin synthesis pathway. Given that molting and reproduction are tightly coupled processes in crustaceans (Waddy et al., 2017), disruptions in chitin biosynthesis within the testis may reduce the systemic availability of chitin precursors, thereby impairing exoskeletal regeneration during infection.

We found that the expression of *phosphoenolpyruvate carboxykinase cytosolic [GTP]-like* gene was downregulated in the intestine of lobsters with ESD. The intestine, as the first line of defense, is one of the main sites of microbial and parasitic infections and serves as a critical organ that requires an efficient and specific immune response to eliminate invading pathogens (Du et al., 2016; Su et al., 2024). Phosphoenolpyruvate carboxykinase catalyzes the conversion of oxaloacetate to phosphoenolpyruvate within the gluconeogenic pathway, thereby playing a key role in maintaining glucose homeostasis (Jane et al., 2024; Sudhakaran et al., 2022; Xing et al., 2025; Yu et al., 2021). For example, increased activity of phosphoenolpyruvate carboxykinase enhances glucose production, which can exacerbate diabetes, whereas enzyme deficiency impairs gluconeogenesis, leading to hypoglycemia (Yu et al., 2021). In addition, phosphoenolpyruvate carboxykinase has been implicated in cold tolerance in lobsters (Farhadi et al., 2023). Thus, the downregulation of phosphoenolpyruvate carboxykinase in lobsters with ESD may indicate impaired glucose production and reduced capacity for stress adaptation.

The HP, also known as the digestive gland, is a major organ involved in energy metabolism, digestion, and nutrient storage, and is known to play an important role in various physiological processes such as stress adaptation and immune response (Farhadi et al., 2023; Ren et al., 2019; Roszer, 2014; Xin et al., 2024; Zhou et al., 2017). Therefore, the gene expression profile of the HP is considered an ideal indicator of stress, immune, and metabolic status (Xin et al., 2024). We found that the expression of the *heat shock protein* (HSP) *70 kDa* genes was significantly downregulated in the HP of lobsters with ESD. HSPs are important for the maintenance of protein homeostasis in cells (Junprung et al., 2021) and play a role in cellular processes during and after exposure to oxidative stress caused by harmful environmental and microbial agents (Kumar et al., 2022). Thus, a decrease in these genes may reflect dysregulation of the cellular stress response. We also found that hemocyanin gene expression was significantly downregulated in the HP of lobsters with ESD. Hemocyanin is a copper-containing protein responsible for oxygen binding, transport, and storage in the blood of invertebrates, and performs several additional functions, including phenoloxidase-like activity (melanization, cuticle hardening), hormone transport, ecdysis, and blood clot formation (Coates and Costa-Paiva, 2020; Kuballa and Elizur, 2008). Hemocyanin also functions as a non-specific immune protein and is an essential component of innate immunity in aquatic invertebrates (Coates and Talbot, 2018; Ji et al., 2024). Together, the significant downregulation of hemocyanin gene expression in the HP may reflect impaired immune function and reduced shell integrity, possibly increasing susceptibility to bacterial infection.

The GG, also known as the antennal gland, is an excretory system responsible for maintaining water and mineral homeostasis, osmotic stabilization, ion regulation, and nitrogen excretion (Buranajitpirom et al., 2010; Khodabandeh et al., 2005; Kruangkum et al., 2024; Ross et al., 2019; Tsai and Lin, 2014). It has recently been shown to act as a natural entry point for viruses and bacteria and has also been implicated in the excretion of metabolites such as molting hormone (De Gryse, 2021; De Gryse et al., 2020). Supporting this role as an entry point, carapace bacteria, including *Aquimarina, Halocynthiibacter*, and *Tenacibaculum*, have been more frequently detected in the GG than in other organs, particularly in lobsters with ESD (Schaubeck et al., 2023). In the present study, we observed significant downregulation of genes encoding HSPs in the GG of lobsters with ESD, consistent with findings in the HP. In addition, genes encoding organic cation transporter proteins were also significantly downregulated in the GG. In humans, organic cation transporter one is primarily expressed in the intestines and liver, while transporter two is highly expressed in the kidneys (Motohashi et al., 2002; Nies et al., 2009; Zhang et al., 1997). Notably, the downregulation of these transporters has been associated with various cancers (Heise et al., 2012; Visentin et al., 2018). Therefore, the reduced expression of these transporter genes in the GG of lobsters suggests a potential disruption of excretory and detoxification functions, which may contribute to disease progression. Chitin, a major component of the exoskeleton, is composed of N-acetylglucosamine (Rowley and Coates, 2023; Zhang et al., 2021), which requires specific transporters for intracellular delivery. In this study, we identified downregulation of genes encoding UDP-xylose and UDP-N-acetylglucosamine transporters in the GG of lobsters with ESD. This downregulation may impair the transport of N-acetylglucosamine, thereby compromising chitin synthesis and exoskeletal integrity.

ESD primarily manifests as a localized infection of the shell, and lobsters may be able to overcome the disease by shedding the infected shell through molting. However, in our study, we observed a significant reduction in the expression of the *estradiol 17β-dehydrogenase 2-like* gene in both the HP and GG of lobsters with ESD. This gene is known to play a role in the metabolic regulation of molting in crustaceans. Previous studies have reported that 17β-estradiol is found in the HP and molting gland of crustaceans (Fu et al., 2022; Liu et al., 2021) and is involved in regulating molting-related processes (Fu et al., 2022; Head et al., 2019). In addition, RNA interference of *estradiol 17β-dehydrogenase* in the HP of *Scylla paramamosain* resulted in a significant decrease in shell hardness (Fu et al., 2022), further supporting the gene’s role in maintaining exoskeletal integrity. Together, these findings suggest that reduced expression of 17E2DH may impair molting, preventing the lobster from shedding the infected shell. This molting failure or delay could exacerbate the infection and potentially lead to systemic disease progression (Schaubeck et al., 2023). Therefore, the downregulation of the *estradiol 17β-dehydrogenase 2-*like gene serves as an important molecular indicator of impaired molting and disease severity in lobsters with ESD.

Our transcriptomic analysis revealed gene expression changes across multiple tissues in lobsters with ESD, highlighting enhanced chitin degradation, suppressed energy metabolism, and reduced cellular stress responses and immune defense. These results provide valuable insight into systemic host responses and the molecular mechanisms underlying disease progression. By uncovering ESD-related genes and tissue-specific expression patterns, this study enables the identification of potential diagnostic markers for the early detection of disease onset. These markers can also support the selection of disease-resistant stocks, contributing to more effective population management and improved long-term health of lobster populations. However, this study is limited by the incomplete gene annotation of *H. americanus*. Among the analyzed DEGs, some are uncharacterized genes with unknown functions, making it difficult to fully interpret their biological significance. Despite this limitation, our analysis identified key functional pathways involved in ESD and provides new evidence that ESD may be driven not only by localized shell infection but also by systemic dysfunction across internal organs. Continued efforts to improve genome annotation and functional characterization of genes in this species will be essential for bridging knowledge gaps in ESD pathogenesis and for developing effective strategies for disease management.

## Data Availability

BioSample metadata have been deposited in the NCBI BioSample database (http://www.ncbi.nlm.nih.gov/biosample/) under accession numbers SAMN48892171-SAMN48892223.
